# The deubiquitinating enzyme USP15 stabilizes ERα and promotes breast cancer progression

**DOI:** 10.1038/s41419-021-03607-w

**Published:** 2021-03-26

**Authors:** Xiaohong Xia, Chuyi Huang, Yuning Liao, Yuan Liu, Jinchan He, Zhenlong Shao, Tumei Hu, Cuifu Yu, Lili Jiang, Jinbao Liu, Hongbiao Huang

**Affiliations:** 1 Affiliated Cancer Hospital & institute of Guangzhou Medical University, Guangzhou, 510095 China; 2 Guangzhou Municipal and Guangdong Provincial Key Laboratory of Protein Modification and Degradation, School of Basic Medical Sciences, Guangzhou Medical University, Guangzhou, 511436 China

**Keywords:** Breast cancer, Translational research

## Abstract

Breast cancer has the highest incidence and mortality in women worldwide. There are 70% of breast cancers considered as estrogen receptor α (ERα) positive. Therefore, the ERα-targeted therapy has become one of the most effective solution for patients with breast cancer. Whereas a better understanding of ERα regulation is critical to shape evolutional treatments for breast cancer. By exploring the regulatory mechanisms of ERα at levels of post-translational modifications, we identified the deubiquitinase USP15 as a novel protector for preventing ERα degradation and a critical driver for breast cancer progression. Specifically, we demonstrated that USP15 promoted the proliferation of ERα^+^, but not ERα^-^ breast cancer, in vivo *and* in vitro. Meanwhile, USP15 knockdown notably enhanced the antitumor activities of tamoxifen on breast cancer cells. Importantly, USP15 knockdown induced the downregulation of ERα protein via promoting its K48-linked ubiquitination, which is required for proliferative inhibition of breast cancer cells. These findings not only provide a novel treatment for overcoming resistance to endocrine therapy, but also represent a therapeutic strategy on ERα degradation by targeting USP15-ERα axis.

## Introduction

Breast cancer (BC) is the most prevalent cancer in women^1^. On the basis of various molecular markers, the breast cancer is classified into estrogen receptor (ER) positive, progesterone receptor (PR) positive, human epidermal growth factor 2 (HER2/ERBB2) positive and triple-negative^2^. Among these receptors, the ER is expressed in approximately 70% of BCs and recognized as the most important therapeutic target for curing this disease^3^. Clinically, the ER-expressing cases can be treated with endocrine therapies, including tamoxifen (a selective ER antagonists), fulvestrant (an ER expression modulators), and letrozole (an aromatase inhibitor)^2^. Although these chemicals displayed a high selectivity and potent inhibitory activities in treating ER^+^ BC, there are growing patients with BCs eventually gain resistance to the therapy. Therefore, it is urgent and necessary to elucidate the molecular regulatory mechanisms of ERα underlying the occurrence of resistance to endocrine therapies and explore an advanced therapy against BC.

Structurally, there are two main forms of the ER, ERα, and ERβ. In BC, estrogen can raise a series of biological effects through these two receptors. At the same time, the clinical data and basic research have reported that estrogen contributes to the development of BC^4^. ERα, a hormone-regulated transcription factor, is expressed as a nuclear receptor in breast epithelial cells. The drug therapy targeting ERα is the mainstay of breast cancer treatment^5,6^. Estrogen can raise many cellular effects through kinds of mechanisms. The classical one is estrogen binds to the ERα, which is located in the nucleus, resulting in the changes of relevant mRNA levels and the production of associated protein, and leading to a biological response^4^. Most of the ERα-triggered effects are mediated by the transcription of ESR1^7^. Moreover, some post-translational modifications, such as ubiquitination, sumoylation, neddylation, and phosphorylation, are involved in the regulation of ERα protein stability or transcriptional activity^8–10^. It has also been demonstrated the ubiquitin-26S proteasome system participates in the regulation of ERα cellular level and transcriptional activity^10^. ERα could be a substrate for several E3 ubiquitin ligase, such as BRCA1/BARD1, MDM2, CHIP, and EFP^11–14^. Deubiquitinases (DUBs), a class of protease to remove the ubiquitin from substrate proteins, play a critical role in regulating protein stability. In our previous study, we have reported that USP7, as a DUB, stabilizes ERα through removing ubiquitin on ERα^15^. A recent study has been reported a deubiquitnase UCHL1 conversely correlated with ERα, suggesting UCHL1 is not a DUB of ERα^16^. Hence, we are prompted to seek other DUBs of ERα.

Ubiquitin carboxyl-terminal hydrolase 15 (USP15), a member of DUBs, belongs to the ubiquitin-specific protease family^17^. USP15 acts as a multifunctional DUB involving in multiple biologic mechanisms by regulating specific substrates. It has also been reported that USP15 is a crucial regulator which is associated with cancer-relevant pathways. Interestingly, USP15 has a dual function in the regulation of cancer progression. As a tumor promoter, USP15 deubiquitinates and stabilizes MDM2, type I TGF-β receptor (TβR-I), and regulates p53 response in cancer cells^18,19^. As a tumor suppressor, USP15 suppresses the cell growth in glioblastoma by stabilizing HECTD1^20^. Our current study intends to uncover the function of USP15 in the development of BC. Further investigations clarified that USP15 is a DUB of ERα protein.

## Results

### USP15 knockdown suppresses the growth of ERα^+^ BC cells

**Fig. 1:**
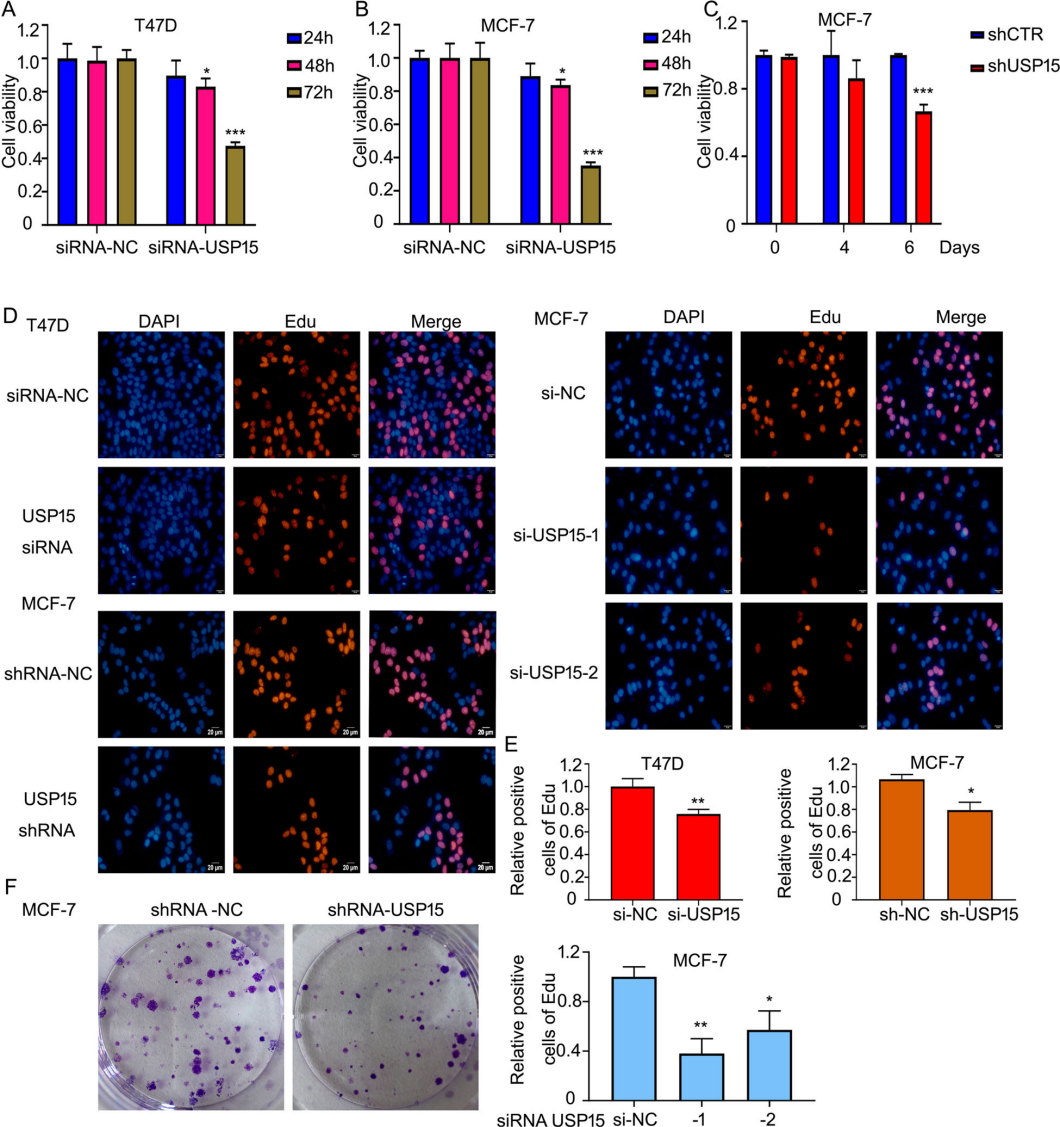
USP15 knockdown suppresses the growth of ERα^+^ BC cells. **A**, **B** T47D and MCF-7 cells were treated with USP15 siRNA for 24, 48, and 72 h. **C** MCF-7 cells stably expressing USP15 shRNA were cultured in 96-wells plates for 4 and 6 days. 20 μl MTS was applied to react for this test of cell viability. ^*^p < 0.05, ^**^p < 0.01, ^***^p < 0.001 *versus* each vehicle control. **D** Cells were transfected with USP15 siRNA or MCF-7 cells were transfected with different sets of USP15 siRNA for 48 h. Edu agent was added and subjected to fluorescence microscope. **E** Stained cells by Edu were counted. ^*^p < 0.05, ^**^p < 0.01 *versus* each vehicle control. **f** Colony formation assay was carried out in MCF-7 treated with USP15 shRNA for 10-14 days.

A study published in *Nature Medicine* reported that the USP15 gene is amplified in BC^18^. But rare researches explore the function of USP15 on the growth of BC and its underlying mechanisms, especially in ERα^+^ BC. To assess the effect of USP15 in ERα^+^ BC, two ERα^+^ BC cell lines, MCF-7 and T47D were included in this study. The MTS assay showed that USP15 siRNAs suppressed the cell viability in a time-dependent manner (Fig. 1A, B). In addition to siRNAs, lentiviruses containing USP15 shRNA also inhibited the growth of MCF-7 cells (Fig. 1C). Additionally, we determined the effect of USP15 in different subtypes of BC, including TNBC cells (HCC1937), HER2^+^/ERα^-^ BC cells (SK-BR3), HER2^+^/ERα^+^ BC lines with tamoxifen resistance (BT474) and HER2^+^/ERα^+^ BC cells (MDA-MB361). The results showed that HCC1937, MDA-MB361, SK-BR3 and BT474 cell lines were not as sensitive as ERα^+^ BCa cells (T47D and MCF-7). It is worth noting that the HER2^+^/ERα^+^ breast cancer cells (MDA-MB361) are more sensitivity to USP15 knockdown than HER2^+^/ERα^-^ breast cancer cells (SK-BR3) (Supplementary Fig. 1). All these findings indicate that ERα may be a critical target for USP15 promoted cell proliferation. Meanwhile, EdU (5-Ethynyl-2’-deoxyuridine) assay, a thymine nucleoside analog replace thymine during DNA replication, was further used to detect the proliferation of cells. Images were captured by fluorescence microscope. The results showed that USP15 silence in both T47D and MCF-7 cells significantly reduced the number of EdU-labeled cells and same situation could be observed in MCF-7 cells after knocking down two different segments of USP15 (Fig. 1D, E). Colony formation assay revealed that the deletion of USP15 by transfecting the short-hairpin RNA (shRNA) in MCF-7 cells has a long-term inhibitory effect on MCF-7 cells (Fig. 1F). The above findings suggest that USP15 plays a role in promoting the growth of ERα^+^ BC cells.

### USP15 deletion triggers cell cycle arrest in ERα^+^ BC cells

**Fig. 2:**
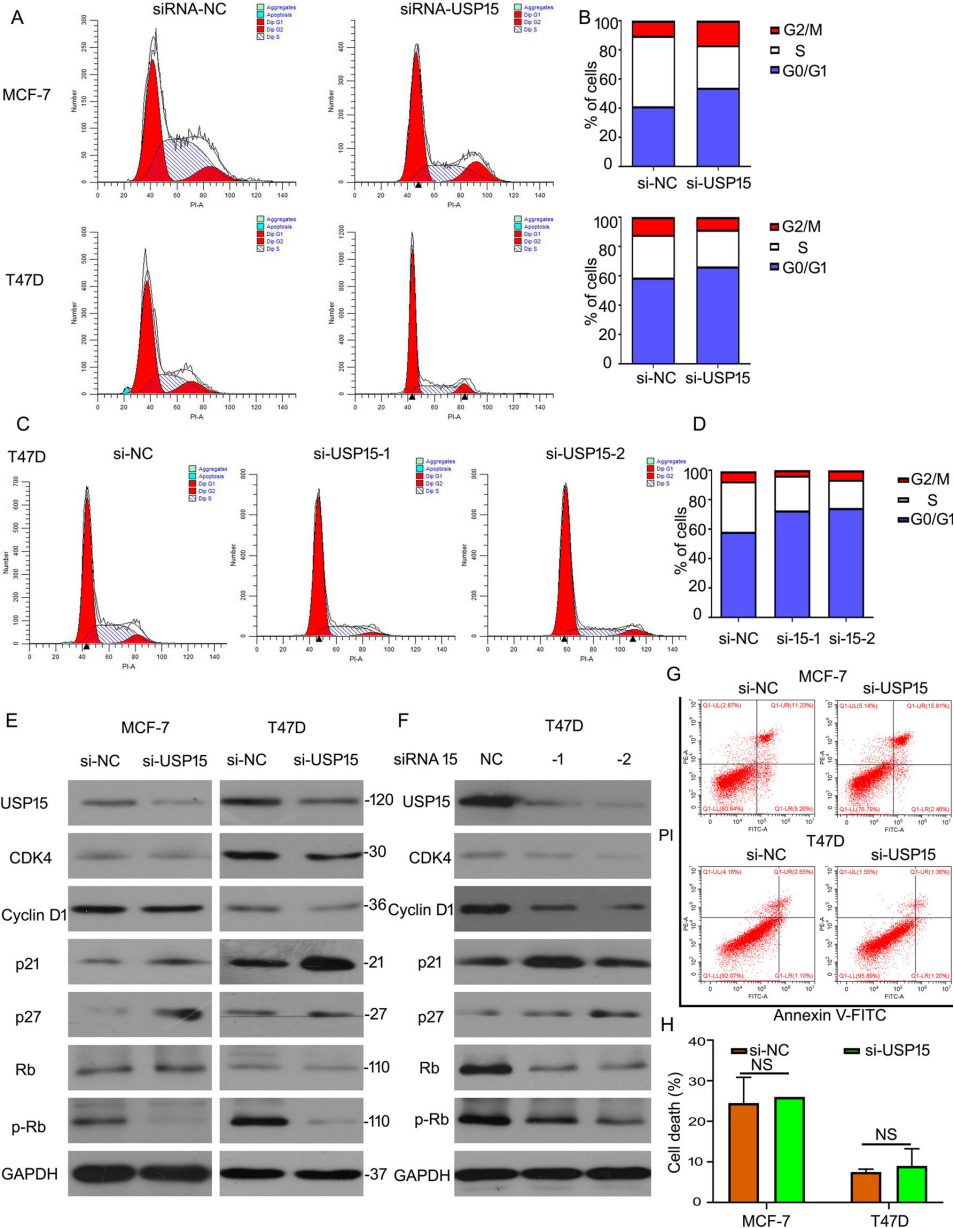
USP15 deletion triggers cell cycle arrest in ERα^+^ BC cells. **A**, **C** Cells were exposed to USP15 siRNA targeting different sets for 48 h, subjected to FACS for cell distributions. **B**, **D** The percent of cell number on each phase was showed. **E**, **F** Cells were treated with USP15 siRNA, proteins were collected for USP15, CDK4, Cyclin D1, Rb, p-Rb, p21, and p27 expression. GAPDH was as a normalizer. **G** The same treated cells were tested using flow cytometry for apoptotic cells and **H** the pooled data has been shown. NS is no significant.

Cell cycle and cell apoptosis are two main pathways in regulating the growth of cancer cells. Given that USP15 knockdown inhibited the progression of ERα^+^ BC cells, we speculated USP15 mediated cell cycle or/and apoptosis in ERα^+^ BC cells. Flowcytometry analysis was used to evaluate cell cycle distribution from G0/G1-G2/M phases. We found that the percentage of cell number was increased at G0/G1 phase post USP15 siRNA treatment in MCF-7 and T47D (Fig. 2A, B). Moreover, to better demonstrate this phenomenon, we purchased two independent sets of siRNAs targeting different regions of USP15 to knock down this gene. Similar results were showed in T47D cells (Fig. 2C, D), indicating USP15 promotes G0/G1 to S phase transition. Then we test the expression of G0/G1 to S phase transition-related proteins, such as CDK4, p-Rb/Rb, Cyclin D1, p21, and p27 in ERα^+^ BC cells after USP15 knockdown. We found that silencing USP15 by three different siRNA induced the downregulation of CDK4, p-Rb/Rb, and Cyclin D1 and upregulation of p21 and p27 in MCF-7 and T47D (Fig. 2E, F). Further cell apoptosis was also performed using flowcytometry analysis. We found that USP15 knockdown did not induce significant cell apoptosis (Fig. 2G, H). These results indicate that USP15 regulates cell growth via the cell cycle pathway, but not cell apoptosis in ERα^+^ BC cells.

### USP15 stabilizes the expression of ERα in BC

**Fig. 3:**
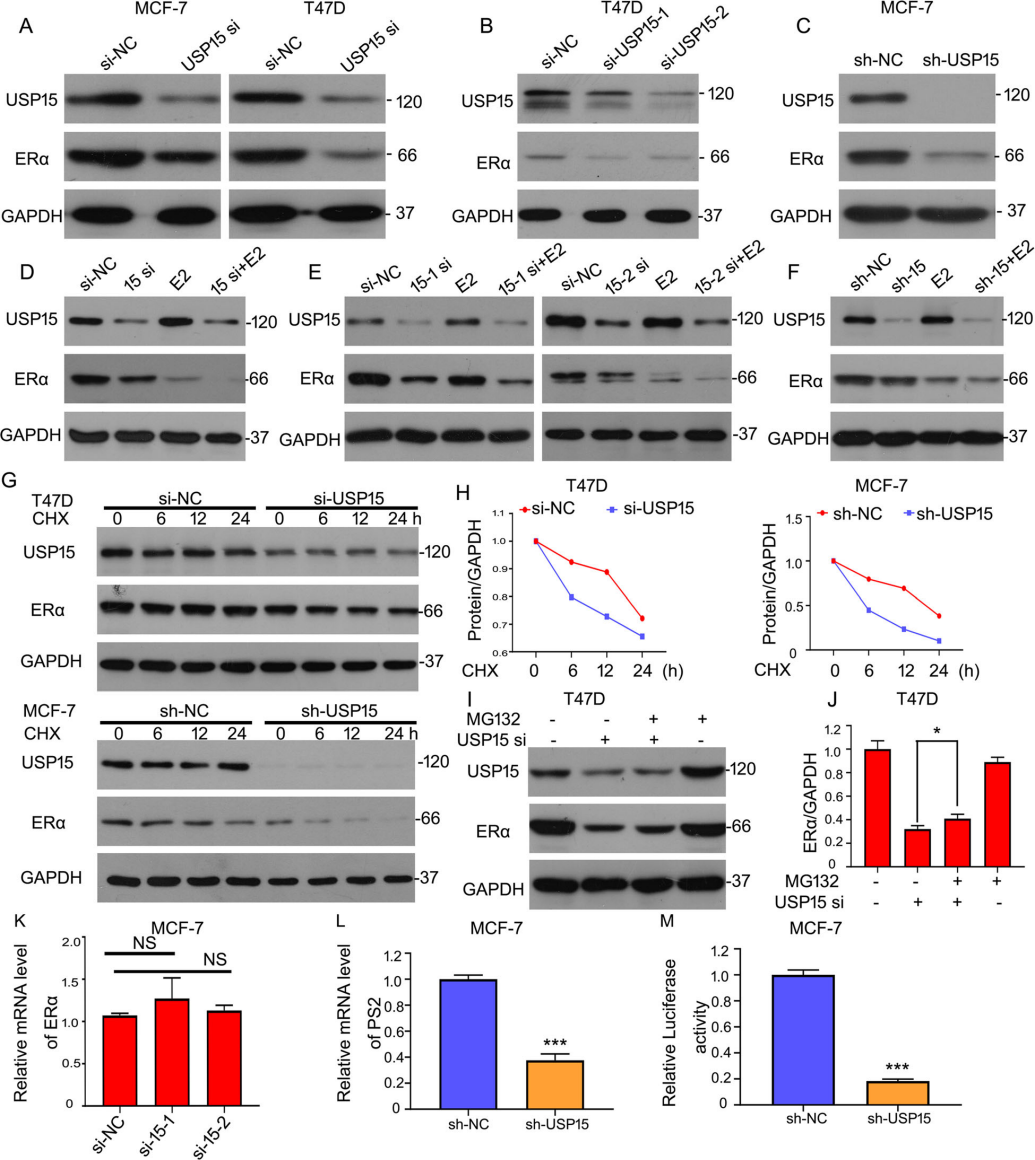
USP15 stabilizes the expression of ERα in BC. **A**–**C** Cells were treated with USP15 siRNA targeting different sets or stably expressing shRNA. Western blot assay for USP15 and ERα expression. **D**–**F** Cells were treated with E2 for 12 h after transfection for 36 h. USP15 and ERα expression were tested. **G** Cells were treated with USP15 siRNA or shRNA and CHX (50 μg/ml). USP15 and ERα expression were evaluated. **H** The value of ERα band was calculated. **I**, **J** USP15 siRNA and MG132 were used to treated with T47D cells, and ERα protein was analyzed by western blot. ^*^p < 0.05. **K** Total RNA were extracted from MCF-7 cells treated with USP15 siRNA targeting different sets, subjected to RT-qPCR for mRNA level of ERα. **L** Total RNA was collected from MCF-7 cells stably expressing shRNA-USP15 and RT-qPCR was applied to evaluate the mRNA level of PS2. **M** Luciferase reporter plasmid containing estrogen receptor elements (EREs) transfected into MCF-7 cells which stably expressing shRNA-USP15 for 6 h. Then medium was replaced by 10% FBS DMEM. Protein lysates were collected, followed by dual-luciferase assay. ^***^p < 0.001.

To clarify molecular mechanisms of USP15 on regulating the growth of ERα^+^ BC cells, we surmised if USP15 plays a role in the expression of ERα. Western blot results showed that the protein level of ERα was remarkably decreased after knockdown of USP15 by transfecting siRNA in the ERα^+^ BC cell lines (MCF-7 and T47D) (Fig. 3A). To eliminate the possibility of off-target effects, two different segments of USP15 siRNA and its short-hairpin RNA (shRNA) were transfected respectively, the expressions of ERα were decreased as shown in Fig. 3B, C. The above results demonstrated that USP15 was positively correlated with ERα in ERα^+^ BC cells. Besides, we also detected the effects of USP15 deletion in the presence of estrogen (E2). Western blot analysis showed that a decrease of ERα was induced by the deletion of USP15 when E2 is existing (Fig. 3D–F). We next explored how USP15 mediated the expression of ERα protein, including suppressing degradation and promoting synthesis. Cycloheximide (CHX), a chemical that inhibits protein synthesis, was used to detect the half-life of the protein. Western blot assay indicated that knockdown of USP15 notably decreased the half-life of ERα proteins (Fig. 3G, H). It has been reported that ubiquitin-proteasome system participated in the degradation of ERα^21^. Thus, we hypothesized that USP15 deletion promoted the degradation through ubiquitin-proteasome pathway. As a result, after blocking the 20 S proteasome activity by treating cells with MG132, the reduced protein level of ERα induced by USP15 knockdown was significantly rescued (Fig. 3I, J), indicating that USP15 is responsible for the stability of ERα protein. Considering the synthesis pathway, the transcription level of ERα and its targeted gene PS2 were tested using RT-qPCR analysis. We found that knockdown of USP15 raised insignificant changes in the mRNA level of ERα (Fig. 3K), but the mRNA level of PS2 was decreased in MCF-7 cells stably expressing shRNA-USP15 (Fig. 3L). Furthermore, to evaluate the ERα-induced transactivation, dual-luciferase reporter assay was performed and showed that the luciferase activity of ERα was suppressed by USP15 shRNA (Fig. 3M).

### USP15 interacts with ERα

**Fig. 4:**
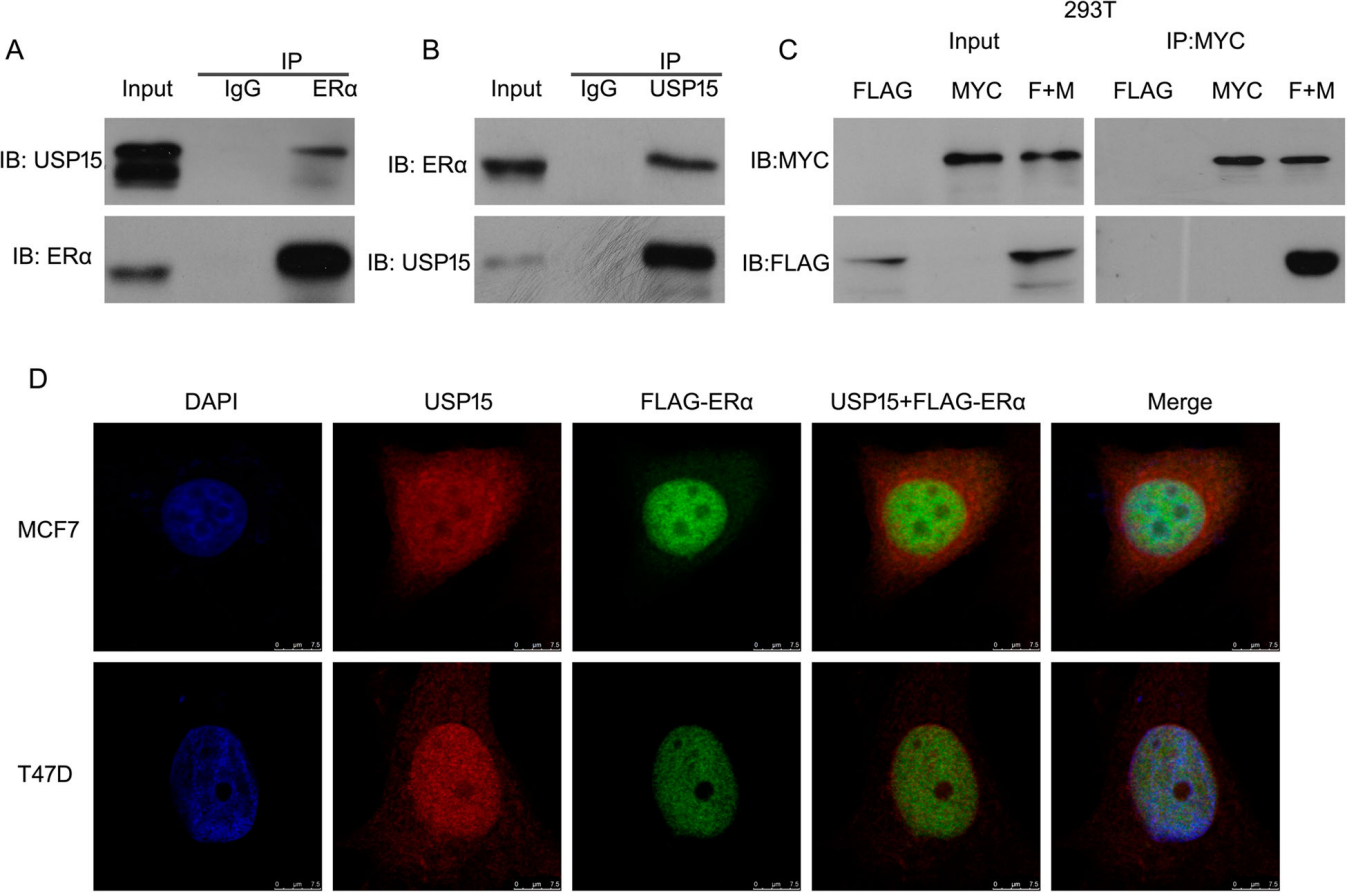
USP15 interacts with ERα. **A**, **B** Proteins were extracted from breast cancer cells. Immunoprecipitated with ERα or USP15, immunoblot with USP15 or ERα. **C** Proteins from HEK293T cells treated with FLAG-tagged ERα and MYC-tagged USP15. Co-IP and western blot assay were applied for MYC and FLAG. **D** MCF-7 and T47D cells were treated with FLAG-tagged ERα and then incubated with anti-USP15 and anti-FLAG antibodies. Images were captured by fluorescence microscope.

The above results demonstrated that USP15 mediates the degradation of ERα protein, suggesting ERα may be a substrate protein of USP15. Hence, we evaluated the potential interaction between the two proteins. Co-immunoprecipitation (Co-IP) with antibodies against ERα followed by immunoblot (IB) with antibodies against USP15 showed that endogenous USP15 notably interacted with ERα, and vice versa (Fig. 4A, B). Moreover, we purchased the plasmids of FLAG-tagged ERα and MYC-tagged USP15 to test exogenic protein interaction. The results of Co-IP assay confirmed the efficient interaction between USP15 and ERα protein (Fig. 4C), which is consistent with the previous results. Immunofluorescence images caught by confocal showed that endogenous USP15 co-localized with FLAG-tagged ERα in the nucleus of MCF-7 and T47D cell (Fig. 4D).

### USP15 promotes deubiquitination of ERα

**Fig. 5:**
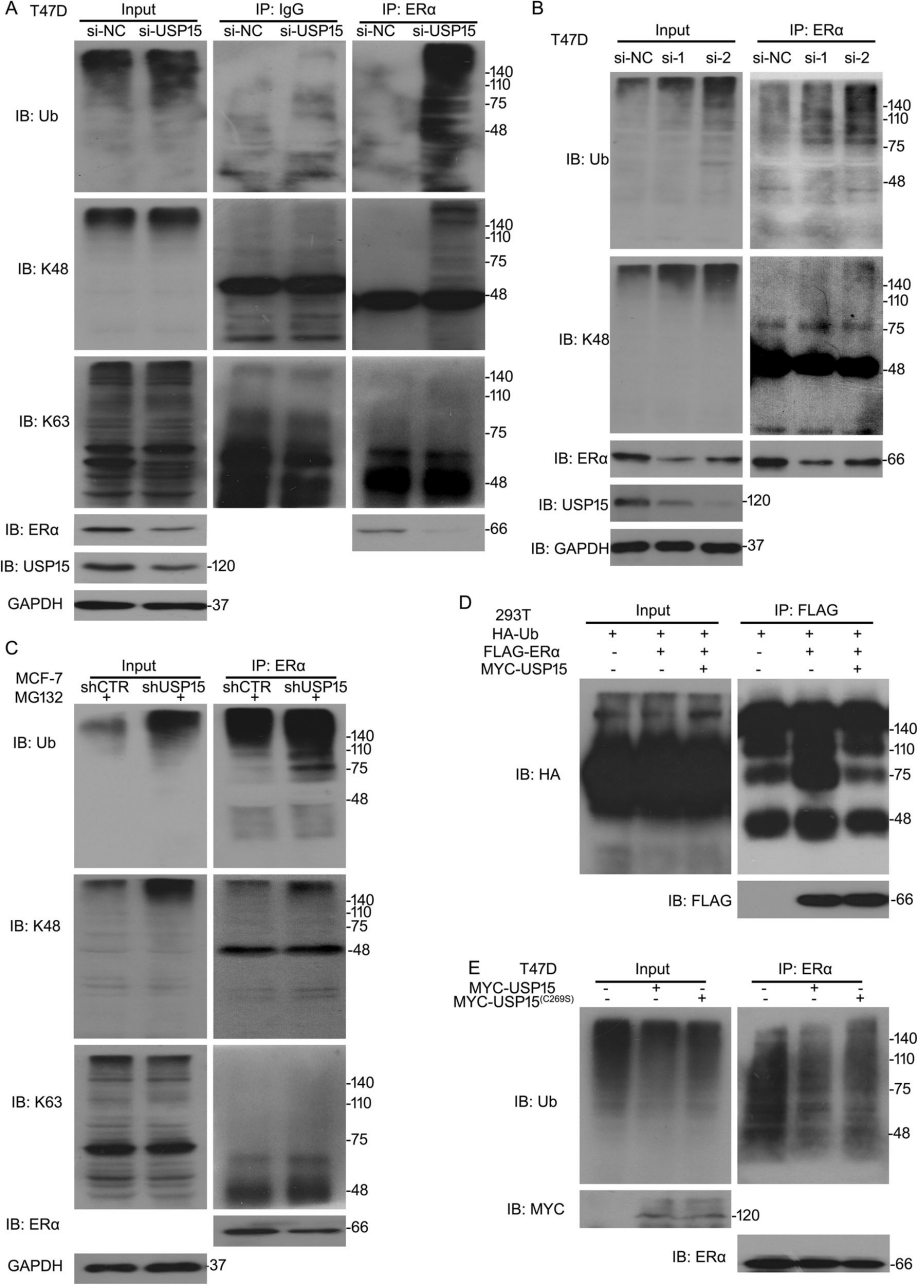
USP15 promotes deubiquitination of ERα. **A** T47D cells were exposed to USP15 siRNA. **B** T47D cells were treated with USP15 siRNA-1, -2. **C** MCF-7 cells were exposed to USP15 shRNA. Proteins were extracted from the above-treated cells. Immunoprecipitated with ERα beads and then immunoblotted with ubiquitin (Ub), K48, ERα, K63. **D** Cells were treated with MYC-USP15, HA-Ub, and FLAG-ERα. Immunoprecipitated with FLAG and then immunoblotted with HA. **E** T47D were transfected with MYC-tagged USP15 mutant (C269S), USP15 (WT). Immunoprecipitated with ERα and then immunoblotted with Ub.

It has been reported that the hinge region K302, K303 of ERα is related to its degradation by UPS^21^. USP15, as a deubiquitinase, stabilizes its substrates through promoting their deubiquitination. Several E3 ligases are responsible for ERα stability. DUBs which protect ubiquitinated ERα escaped from degradation were less to know. Given that USP15 is a regulator on the degradation of ERα protein, we wonder if USP15 is a meritorious DUB for ERα. To unlock the puzzle, we performed the Co-IP analysis to test the level of poly-ubiquitinated ERα. Results showed that knockdown or knockout of USP15 notably increased the expression of ubiquitinated ERα in the ERα^+^ BC cells (Fig. 5A–C). Besides, our results demonstrated that deletion of USP15 potentiated K48-linked ubiquitination of ERα, but did not affect the K63-linked ubiquitination(Fig. 5A–C), suggesting USP15 silence promotes degradation of ERα through K48-linked ubiquitination. To further confirm whether USP15 could affect the ubiquitination of ERα, HEK293T cells were overexpression of wild-type USP15 (USP15/WT). Co-IP assay showed that overexpression of USP15/WT decreased the level of ubiquitination of ectogenous ERα (Fig. 5D). T47D cells expressing MYC-tagged catalytically inactive mutant of USP15 (USP15/C269S) or MYC-tagged USP15 were established. The abundance of poly-ubiquitinated ERα was more in USP15 (C269S) group than that of USP15 (WT) (Fig. 5E), indicating that the enzymatic activity of USP15 is required for the deubiquitination of ERα.

### USP15 promotes the growth of ERα^+^ BC cells depends on ERα status

**Fig. 6:**
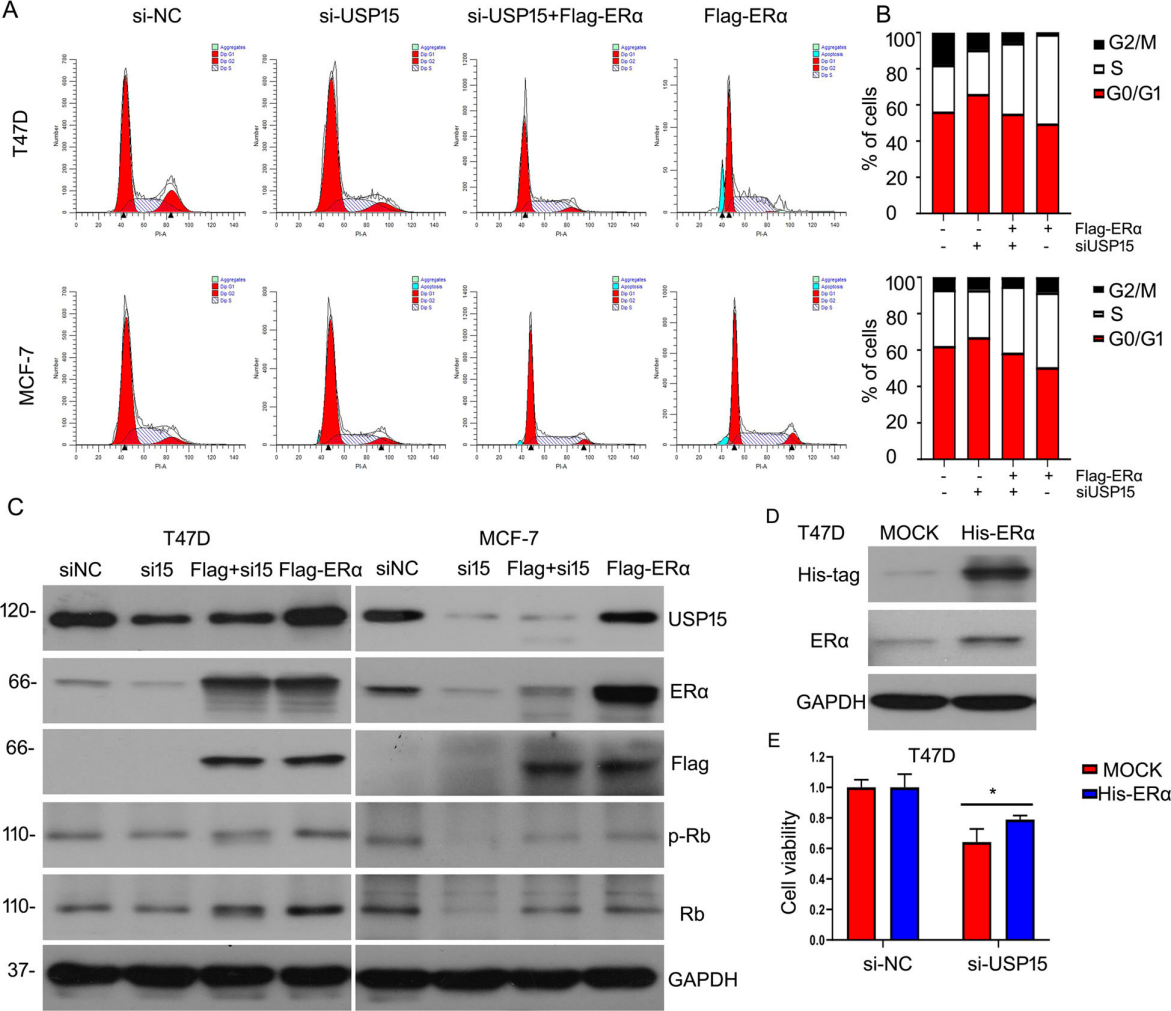
USP15 induces the growth of ERα^+^ BCcells depends on ERα status. **A**, **B** Cells were treated with USP15 siRNA and FLAG-tagged ERα, followed by FACS. Cells distribution also was showed. **C** Proteins were subjected to western blot for ERα, USP15, FLAG, Rb, p-Rb. **D** Cells were transfected with His-tagged ERα lentivirus. Protein was collected for western blot to test His and ERα expressions. **E** Cells stably overexpressing ERα were treated with USP15 siRNA in 96-well plates. MTS was applied to react to this test of cell viability. ^*^p < 0.05 *versus* each vehicle control.

We have confirmed that USP15 silence inhibited cell cycle progression to suppress BC progression and USP15 stabilized the protein expression ERα in ERα^+^ BC cells. However, whether the two aspects of effects induced by USP15 exists a correlation is not be clarified. We overexpressed ERα protein using FLAG-tagged ERα under USP15 deletion treatment. Flowcytometry analysis was applied to evaluate cell cycle distribution. The results showed that overexpressing ERα partly abrogated cell cycle arrest induced by silencing USP15 in MCF-7 and T47D cells (Fig. 6A, B). Western blot analysis was carried out to verify the efficiency of transfecting full-length plasmid of ERα and USP15 siRNA. We observed the expression of ERα protein was overexpression and USP15 is decreased. Importantly, the expression of proteins related to cell cycle was rescued by ERα under USP15 siRNA treatment (Fig. 6C). To further investigate the relationship between USP15 and ERα, we tested cell viability using MTS assay. Firstly, we transfected lentivirus carrying His-ERα or control vector (MOCK). Then cell was treated with USP15 siRNAs. The inhibitory effect of cell growth induced by USP15 siRNA was rescued by overexpressing ERα protein(Fig. 6D, E). These results suggesting ERα expression is indispensable to USP15 knockdown-induced inhibitory effect on the growth of BC cells.

### USP15 overexpressed in breast cancer and USP15 silence inhibits cancer progression in MCF-7 xenograft

**Fig. 7:**
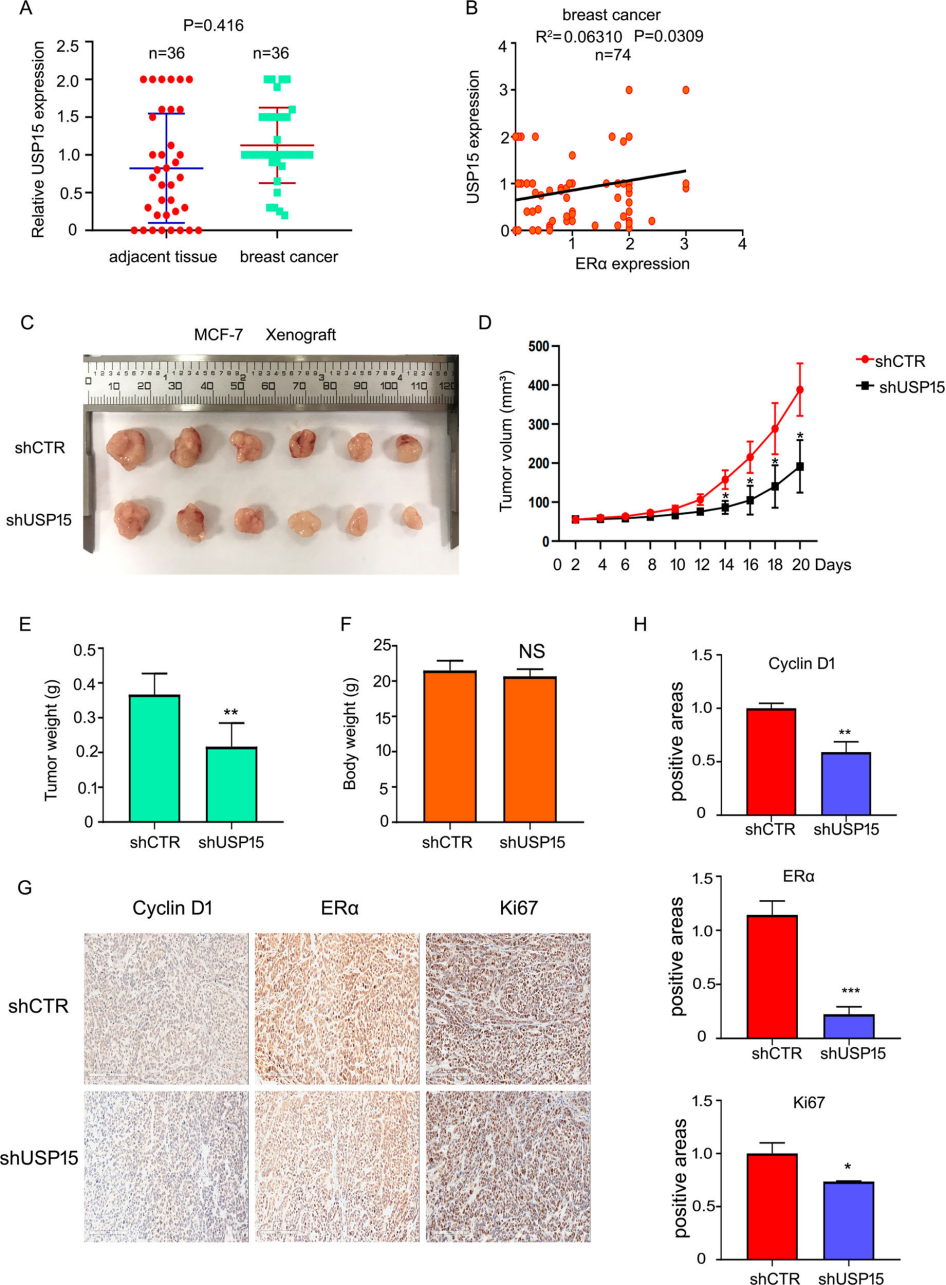
USP15 silence inhibits cancer progression in MCF-7 xenograft. **A** Expression of USP15 in human breast cancer tissues (*n* = 36) and adjacent tissues (*n* = 36). **B** The correlation of USP15 and ERα levels (*n* = 74). **C** Tumor images of mice with injected of MCF-7 cells stably expressing USP15 shRNA. **D** Tumor volume, **E** tumor weight and **F** body weight were showed. *p < 0.05, **p < 0.01 *versus* each vehicle control. **G**, **H** ERα, Ki67, Cyclin D1 expression were tested using immunohistochemical assay. *p < 0.05, ***p < 0.001 *versus* each vehicle control.

To further identify the role of USP15 in breast cancer, we evaluated its expression using clinical samples from patients with breast cancer. The results of immunohistochemistry assay showed that USP15 expression was higher in human breast tumor tissues than in adjacent tissues (Fig. 7A). In accord to staining intensity, we also found that USP15 protein expression was positively correlated with that of ERα (Fig. 7B). The function of USP15 on ERα^+^ BC in vitro has been demonstrated. We sought to determine whether USP15 also play a role in vivo. Xenografts in vivo have been established using USP15 shRNA stably expressing MCF-7 cells. After 20 days, we found that the volumes of xenograft were shrunken in the group of USP15 shRNA (Fig. 7C, D). Tumor weight was lighter in USP15 shRNA-treated group than that of scramble shRNA-treated group (Fig. 7E). However, the weight of body was nearly equal (Fig. 7F). In addition, we also test protein expression of Cyclin D1, ERα and Ki67 using IHC analysis. The results showed that the expressions of these proteins were decreased (Fig. 7G, H). These results indicated that USP15 suppressed ERα^+^ BC growth not only in vitro, but also in vivo.

### USP15 knockdown enhances the sensitivity of ERα^+^ BC cells to tamoxifen

**Fig. 8:**
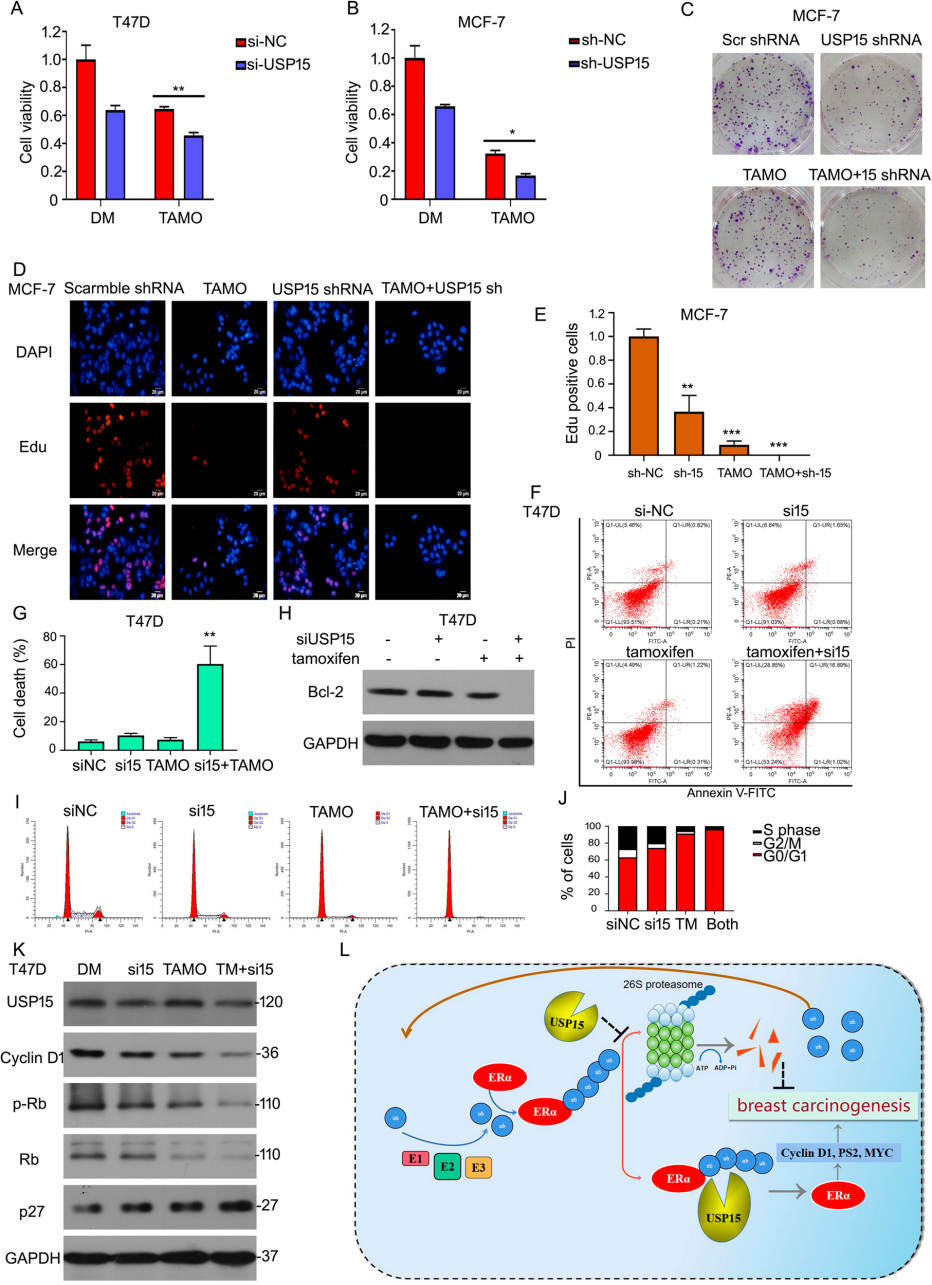
USP15 knockdown enhances the sensitivity of ERα^+^ BC cells to tamoxifen. **A**, **B** Cells were exposed to USP15 siRNA/shRNA and tamoxifen (TAMO, 20 μM/L). Cell viability was detected by adding MTS. **C** Colony formation assay was done. **D**, **E** EdU staining assay was done and images were captured by fluorescence microscope. ^*^p < 0.05, ^**^p < 0.01, ^***^p < 0.001 *versus* each vehicle control. **F** T47D cells were treated with tamoxifen, USP15 siRNA or the combination of the two drugs. Cell apoptosis was tested by flow cytometry for apoptotic cells. **G** The pooled data has been showed. ^**^p < 0.01 *versus* each vehicle control. **H** And protein was collected for western blot to evaluate Bcl-2 expression. **I** The treated cells were subjected to FACS for cell distributions. **J** The percent of cell number on each phase was showed. **K** Proteins were extracted from T47D treated with USP15 siRNA and tamoxifen, subjected to western blot for Cyclin D1, Rb, p-Rb, p27 expression. **L** A proposed mechanism for USP15 to regulate the ERα level in breast cancer.

Tamoxifen is an antagonist of estrogen through binding to ER**α** and is applied to clinical therapy in patients with BC. However, drug tolerance decreases the anti-tumor effects of tamoxifen. We explore if USP15 can improve the anti-tumor function of tamoxifen in ERα^+^ BC cells. MTS agent was used to test cell viability under USP15 siRNA/shRNA or tamoxifen treatments. The results showed that the combination treatment of silencing USP15 expression and tamoxifen induced more obvious inhibitory effects in MCF-7 and T47D cells (Fig. 8A, B). Additionally, the inhibition of long-proliferation was also more in the combination of two treatment using colony formation analysis (Fig. 8C). EdU staining analysis showed that USP15 shRNA increased anti-proliferative effect of tamoxifen (Fig. 8D, E). Moreover, we test cell apoptosis and cycle assays to test the synergistic effects of USP15 and tamoxifen. These results showed that cell apoptosis was induced by USP15 siRNA + tamoxifen treatment (Fig. 8F–H). USP15 silence enhanced tamoxifen induced-cell arrest at G0/G1 phase (Fig. 8I, J). Some proteins involving in cell cycle progression, such as Cyclin D1, p-Rb/Rb, p27, were tested using western blot analysis. We found that the decreased expression of Cyclin D1 and p-Rb/Rb and increased expression of p27 were more advanced by adding tamoxifen to USP15 siRNA treatment (Fig. 8K). These results indicate that silencing USP15 suppresses cell growth of BC in part through promoting ERα degradation (Fig. 8L).

## Discussion

The high incidence and mortality of BC have become a threat to women’s health all over the world. Endocrine therapy targeting hormone receptor is the main scheme in patients with BC. Owning to approximately 2/3 of BC expresses estrogen receptor alpha (ERα), tamoxifen or fulvestrant, modulators aiming at ERα, play the most effective prognosis in the treatment of BC. But some patients developed resistance to endocrine therapy at a later stage, resulting in relapse and even migration^22–24^. Now that resistance is inevitable, new strategy should be put forward urgently.

The function of endocrine therapy is mainly blocking the activity of ERα via disturbing the binding between ERα and estrogen. But promoting the degradation of ERα protein in the therapy of BC has not been well understood and applied to clinical trial. Ubiquitin-proteasome pathway (UPS) is involved in the degradation of ERα protein^21,25,26^. Deubiquitinase (DUB) expression generally is high in multiple cancers and promoting tumor progression. Meanwhlie, some researches have been reported that DUBs can directly regulate the function of a number of oncoproteins. Oncoproteins are their substrates. For example, USP14 interacts with androgen receptor (AR) to regulate the progression in prostate cancer and breast cancer^27,28^. USP7 mediates the stability of PHF8^29^. USP9X control EGFR fate by deubiquitinating endocytic adaptor Eps15^30^. The DUB of ERα has not been deeply explored. Until now, the first DUB (USP7) of ERα is found in our study^15^. Whether other DUB of ERα exists is unknown. There is a report indicates that the level of USP15 is amplified by 1.8% in BC tissues from the patients with BC^18^. USP15 is a DUB. We ask a question curiously, whether USP15 indeed makes an impact on the degradation of ERα. In this study, we suggested that USP15 stabilizes the expression of ERα protein via inhibiting its degradation and promotes cell growth of BC.

The role of USP15 has been demonstrated in several cancer development^31–33^. Here, we confirmed the control of USP15 in the growth and progression of ERα positive BC (ERα^+^ BC). MTS and EdU staining assays showed that USP15 deletion significantly induces the growth inhibition of ERα^+^ BC cells. The long-proliferation inhibition also be triggered by silencing USP15. Growth suppression mainly resulting from the cell cycle arrest, not cell apoptosis induced by USP15 knockdown. In addition to in vitro, USP15 silence blocks xenograft growth in vivo while the bodyweight of mice is not significantly decreased. Considering the relapse of BC due to tamoxifen resistance, we speculated USP15 may be increased the anti-cancer effect of tamoxifen in BC cells. Then we employ MTS, EdU, western blot, and colony formation assays to evaluate cell proliferation under the combined treatment of USP15 deletion and tamoxifen. The inhibitory function is more obvious, suggesting USP15 maybe enhance the sensitivity of ERα^+^ BC to tamoxifen to reduce the relapse because of drug resistance.

We further study the molecular regulatory mechanisms of USP15. USP15 remarkably mediates the protein expression of Cyclin D1 and p21. As known, the two molecular are targeted genes of ERα^34^. Hence the regulation of ERα by USP15 is possibly contributing to cell cycle progression. Western blot assays verified that decreased ERα protein expression is induced by silencing USP15 in ERα^+^ BC cells. Moreover, we found that USP15 plays a role in the degradation of ERα. MG132, 20 S proteasome inhibitor, abrogates partly decreased ERα expression by USP15 knockdown, suggesting USP15 stabilizes ERα expression through 20 S. The mechanism of degradation further is clarified that USP15 deletion increases the ploy- and K48-ubiquitination on ERα. Moreover, the results of Co-IP assay show that USP15 interacts with ERα. Significantly, overexpressing ERα protein inhibits cell cycle arrest triggered by USP15 knockdown. These findings demonstrated that USP15 promotes the proliferation of BC via stabilizing ERα expression (Fig. 8l).

Taken together, this study reveals that USP15 contributes to the expression and stability of ERα protein by regulating its deubiquitination and further promotes the growth of ERα^+^ BC, which may provide a preclinical basis for new therapy for treating BC.

## Materials and methods

### Cell lines and culture method

Human embryonic kidney cell line, HEK293T, and human breast cancer cell lines, T47D, MCF-7, MDA-MB361, SK-BR3, HCC1937, and BT474 were from ATCC (Manassas, VA, USA). HEK293T, MCF-7, BT474, MDA-MB361, and T47D cells were cultured in DMEM, supplemented with fetal bovine serum (FBS) to a final concentration of 10%. MCF-7 cells need to add 10 mg/ml human recombinant insulin. HCC1937 cells were cultured in RPMI 1640 supplemented with 10%. SK-BR3 cells were cultured with McCoy’s 5 A medium with 10% FBS. 37 °C and 5% carbon dioxide (CO_2_) were employed for cell culture.

### Reagents and antibodies

MG132 (S2619), tamoxifen (S1238) were obtained from Selleck (Houston, TX, USA). Control siRNA (sc-37007) and USP15 siRNA (sc-76819) were from Santa Cruz Biotechnology (Santa Cruz, CA, USA). USP15-1 and USP15-2 siRNA (S0222) were from genechem (Shanghai, China). Cell-Light^TM^ EdU Apollo 567 In Vitro Kit (C10310-1) was obtained from RiboBio (Guangzhou, China). Annexin V-FITC/PI apoptosis detection kit (KGA107) was obtained from Keygen Company (Nanjing, China). The antibodies purchased from Cell Signaling Technology (CST, MA, USA) are listed below: anti-p21 (2947), anti-USP15 (66310), anti-ubiquitin (3936), anti-estrogen receptor α (8644), anti-phospho-Rb (8516), anti-Rb (9313), anti-Cyclin D1 (2922)(55506), anti-CDK4 (12790), anti-p27 (3686), anti-GAPDH (5174), anti-K48-ub (12805), anti-K63-ub (12930), anti-DYKDDDDK-tag (14793), anti-Myc-tag (2276). Anti-ERα (ab32063) was from Abcam (USA). Anti-USP15 (67557-1-lg) was from proteintech (Chicago, USA). Lentivirus was purchased from VigeneBio (Shandong, China) and plasmids were from Genechem (Shanghai, China). Cycloheximide (CHX) and Estrogen (E8875) were received from Sigma-Adrich (Sigma-Adrich, Louis, MO). MTS (catalog no. G111) was purchased from Promega Corporation (Madison, WI, USA). Co-IP assay kit (14311D) was purchased from Life Technologies (Carlsbad, CA).

### Cell viability assay

The experiment was carried out as the previous steps which we have been reported^35^. Cells were digested from the flask, resuspended, and evenly seeded into the 96-well plate. After adherent cells were transfected with siRNA for 24 h, 48 h, and 72 h. The OD value was detected by using MTS to estimate cell viability.

### EdU staining

Detecting the cell proliferation, we used Cell-Light™ EdU Apollo 567 In Vitro Kit (Cat number: C10310–1, RiboBio, Guangzhou, China). Cells were planted into the chamber slide before transfected with siRNA for 48 h. After that, 50 μM EdU was used to incubate with cell for 2 h. Briefly, cells were stained with Apollo^®^ fluorescent dye and counterstained with DAPI. The EdU-positive nuclei were captured by Olympus microscope.

### Cell cycle and apoptosis assay

The experiments were carried out according to our previous reports^28,36^. Cells were seeded into 6-well plate, after adherent cells were treated with siRNA for 48 h. Cells were collected by centrifugation. Cells used to detect cell cycle were resuspended with 500 μl PBS and added 2 ml 70% ethanol for fixation and permeabilization overnight. 50 μg/ml PI, 0.2% Triton-X-100, and 100 μg/ml RNase complex were applied in dark. Annexin V- FITC apoptosis analysis kit was purchased (SUNGENE BIOTECH) for test apoptotic cells. The sample was analyzed by flow cytometry.

### Cell clonogenic assay

The assay was done as previously described^37^. Harvest USP15-stably-transfected cells and plated on a 12-well plate, incubated cells for 48 h in a CO_2_ incubator at 37 °C. The same number of cells were randomly plated in 6-well plate, and incubated cells in CO_2_ incubator. After fixed with 4% paraformaldehyde, crystal violet solution staining was performed.

### Immunoblotting

This assay was done as we previously reported^38^. Protein lysate was scraped from the adherent cells and centrifuge in a microcentrifuge at 4 °C. BCA protein assay kit was used to quantify collected proteins. Equal amounts of protein were loaded into the wells of the SDS-PAGE gel. Transferred the separated protein from the gel to the PVDF membrane. Blocked the membrane using defatted milk for an hour. Primary antibody incubates with membrane overnight. The conjugated secondary antibody was added with defatted milk to incubate the membrane for an hour. Detected the expression of indicated protein using ECL detection reagents.

### Real-time polymerase chain reaction analyses

RNAs were collected from MCF-7 cells using RNAiso plus (TaKaRa Biotechnology, Dalian, China) after transfected with siRNA for 24 h. We purified and detected the concentration of RNA. The first-strand cDNA was from the RNA using PrimeScript RT Master Mix kit (TaKaRa, Dalian, China). The mRNA levels of ERα and GAPDH were evaluated using SYBR Premix Ex TaqTM kit (TaKaRa, Dalian, China). PCR primers were as same as that of in this report^15^.

### ER-Positive Xenograft in mice

The female nude mice (18–22 g) were purchased from Sibeifu (Beijing) biotechnology co. LTD and animal protocols were approved by the Institutional Animal Care and Use Committee of Guangzhou Medical University. After inspection and quarantine, mice were placed in barrier facilities. Firstly, 0.72 mg/90-day-release-17β-estradiol pellets were implanted subcutaneously into each mouse for one week. The nude BALB/c mice were randomly separated into two groups and subcutaneously implanted with MCF-7 cells as the control group or MCF-7 cells stably expressing USP15 deletion as the treatment group. During the month of observation, the volume of tumor and the weight of mice were tested every other day.

### Immunohistochemical staining

Assay was performed as the previous study we reported^38^, xenografts harvested from the mice were fixed with 4% paraformaldehyde and embedded in paraffin. A total of 4 μm sections were cut from paraffin-embedded specimens. Sections were subjected deparaffinized with xylene, rehydrated. Keeping in antigen retrieval buffer containing EDTA and microwaved performed antigenic retrieval. Around 1% BSA solution was applied to block nonspecific binding. The sections were stained using anti-Cyclin D1, anti-Ki67, and anti-ERα antibodies overnight at 4 °C. Tissues were incubated with secondary antibody, subjected to diaminobenzidine (DAB) and H2O2 in 50 mM tris-HCl. Hematoxylin was used to counterstain the slides.

### Si/ShRNA and plasmid transfection

The transfection assay was done as reported in our study^39^. BCa cells were seeded into the appropriate plate or dish. For the siRNA transfection assay, RPMI opti-MEM (Gibco), lipofectamine RNAiMax (Invitrogen) reagent, and USP15 or control siRNAs mixtures were prepared. SiRNA mixtures were added to the cells after incubated for 15 min. For lentivirus USP15 shRNA and ERα transfection, the lentivirus, medium, and polybrene (5 μg/ml, Santa Cruz, CA, USA) mixtures were applied to incubate cells and treated for 48 h. Puromycin for USP15 and blast for ERα were for selecting stable-transfected cells. For the plasmid transfection assay, RPMI opti-MEM (Gibco), lipofectamine 3000 (Invitrogen) reagent, and plasmid or control vector mixtures were incubated for 15 min and added to the cells, respectively for 48 h.

### Confocal assay

The BCa cells were planted into the confocal dish. Briefly, 4% paraformaldehyde was applied to fix the treated cells for 15 min, and 0.5% Triton-X for permeabilization. Then 5% BSA was used to block for 30 min, primary antibody was added to treat cell overnight, subjecting to secondary antibody for an hour, then DAPI was added for the staining of cell nucleus. Images were captured by Leica laser confocal microscope.

### Luciferase reporter promoter assay

MCF-7 cells stably expressing shRNA-USP15 were seeded into 24-well plates. Cells were transfected with 10 ng luciferase reporter plasmid containing estrogen receptor elements (EREs). After 6 h, the medium was replaced by 10% FBS DMEM. According to the manufacture’s instructions, dual-luciferase assays kit was applied to test the activity of luciferase. The relative luciferase was counted by firefly lucifersae to Renilla luciferase.

### Patients’ selection

Three tissue arrays were purchased from Shanghai Outdo Biotech Company (Shanghai, China). Tissue arrays were approved by the ethics committee of Shanghai Outdo Biotech Company(Shanghai, China). The adjacent tissue and tumor tissue from 36 patients with breast cancer for USP15 expression. Among these, 74 human breast cancer tissues were from 74 patients for ERα and USP15 expression.

### Statistical analysis

The data are mean ± SD from three independent experiments. Unpaired Students’s *t* test or one-way ANOVA is applied where appropriate to analysis the statistical probabilities. GraphPad Prism8.0 software and SPSS 16.0 are used to perform statistical test. *P* value of < 0.05 is considered as statistically significant.

## Supplementary information

Figure S1

Supplementary Materials
