# Supplementary figures and images for: The deubiquitinating enzyme USP15 stabilizes ERα and promotes breast cancer progression
Source: Cell Death Dis. 2021 Mar 26;12(4):329. doi: 10.1038/s41419-021-03607-w (PMC7997968; doi:10.1038/s41419-021-03607-w)

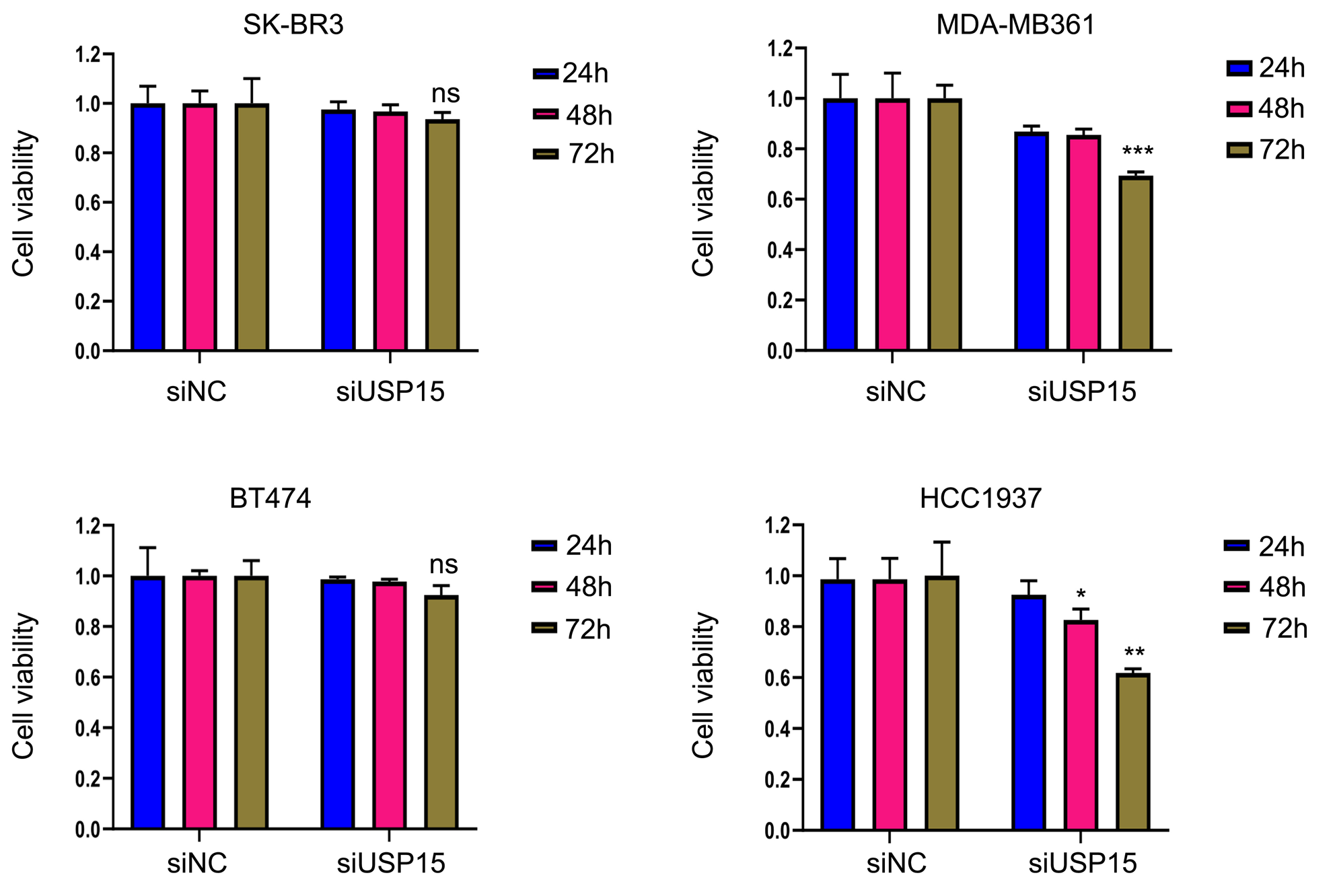

Supplement: Supplementary file 1 — Figure S1 [file 41419_2021_3607_MOESM1_ESM.tif]
