# Supplementary material for: The deubiquitinating enzyme USP15 stabilizes ERα and promotes breast cancer progression
Source: Cell Death Dis. 2021 Mar 26;12(4):329. doi: 10.1038/s41419-021-03607-w (PMC7997968; doi:10.1038/s41419-021-03607-w)
Supplement: Supplementary file 2 — Supplementary Materials [file 41419_2021_3607_MOESM2_ESM.docx]

**Fig. S1** **The role of USP15 on different subtypes of BC.** Cells were treated with USP15 siRNA for 24, 48 and 72 h. 20 μl MTS was applied to react for this test of cell viability. ^*^p<0.05, ^**^p<0.01, ^***^p<0.001 *versus* each vehicle control.
